# Targeting gut dysbiosis: a new frontier in MASLD management

**DOI:** 10.3389/fsysb.2026.1937809

**Published:** 2026-09-25

**Authors:** Shweta Sharma, Arun Pachauri, Harshal Nagar, Vishwa Thakkar, Kajal Patel

**Affiliations:** 1 Department of Pharmacy Practice, Parul Institute of Pharmacy, Faculty of Pharmacy, Parul University, Vadodara, Gujarat, India; 2 Preventive Health and Clinical Services, Deepak Foundation, Vadodara, Gujarat, India

**Keywords:** biotics, chronic liver disease (CLD), fecal matter transplantation (FMT), gut dysbiosis, gut-liver axis, hepatic diseases, metabolic dysfunction-associated steatotic liver disease (MASLD), steatohepatitis

## Abstract

Globally, Metabolic dysfunction-associated steatotic liver disease (MASLD) is a prominent leading cause of CLD, which is intricately linked with obesity, T2D, and metabolic syndrome. Recent insights in pathophysiologically of MASLD have shifted from the traditional “two-hit” model to a “multiple-hit” paradigm, demonstrating the importance of understanding the gut-liver axis and gut dysbiosis as central drivers of disease progression. Hence, this narrative review explores the mechanistic pathways through which an altered gut microbiome contributes to MASLD, by specifically emphasizing intestinal barrier disruption, endotoxemia, altered bile acid metabolism, and SCFA imbalance. These dysregulations trigger oxidative stress, leading to systemic inflammation and insulin resistance, propelling simple steatosis toward steatohepatitis and fibrosis at last. Furthermore, we also highlighted the diagnostic utility of microbial signatures and multi-omics approaches in identifying disease-specific microbial shifts, such as the depletion of beneficial SCFA-producing bacteria like *Faecalibacterium* and *Akkermansia*. Eventually, to address the growing hepatic-associated health burden, microbiome-targeted therapeutics can emerge as one of the promising contributors. In this review, we synthesise and demonstrate the current and prospective interventions, including dietary modifications, biotics (probiotics, prebiotics, synbiotics), and FMT. Additionally, we also show the transition of care toward precision microbiome therapies utilizing engineered bacteria, bacteriophages, and AI-guided patient stratification. While current microbiome-centric treatments demonstrate promise in modulating hepatic inflammation and restoring mucosal integrity, their clinical translation remains hindered by patient heterogeneity and a lack of standardized protocols. Ultimately, managing MASLD effectively will require moving beyond broad ecosystem resets toward highly tailored, multi-omics-driven precision medicine to accurately target individualized microbial deficits.

## 1 Introduction

Non-alcoholic fatty liver disease (NAFLD) has become the most frequent cause of chronic liver disease, affecting nearly 38% of the adult population worldwide (Targher et al., 2024). To accurately reflect the condition’s metabolic background, a multi-society Delphi consensus recently adopted the replacement term metabolic dysfunction-associated steatotic liver disease (MASLD). Under this updated nomenclature, the diagnosis of MASLD is positive and requires the presence of hepatic steatosis in conjunction with at least one of five cardiometabolic risk criteria. Furthermore, this consensus introduced the overarching term of steatotic liver disease (SLD), which includes an intermediate category, MetALD, to appropriately classify patients who meet the criteria for MASLD but also exhibit alcohol consumption exceeding the strict thresholds set for pure MASLD (Rinella et al., 2023). MASLD patients commonly have a strong association with type 2 diabetes (T2D), obesity, cardiometabolic incidents, chronic kidney disease, liver and non-liver cancer, and serious liver-related complications such as metabolic dysfunction-associated steatohepatitis (MASH) (Younossi et al., 2024; European Association for the Study of the Liver, 2024).

In light of the main factor of lifestyle changes being used in the management of MASLD, the development of new drugs for treating it is an urgent matter. The gut-liver axis has been identified as a major area of focus in this search (European Association for the Study of the Liver, 2024; Lau et al., 2025). As an example, the gut microbiota interacts with liver metabolism and inflammation through mechanisms such as the intestinal mucosal barrier and immune response of the host plus microbial metabolites. Hence, gut dysbiosis (a change in the normal composition of gut microbiota) plays an important role in causing this disease rather than just being a witness (Yang et al., 2025; Zhu et al., 2026; Zhang X. et al., 2025; Li et al., 2024; Qian et al., 2025). When it comes to MASLD, the disruption of this delicate system by gut dysbiosis leads to a cascade of negative outcomes. The changes in microbial composition and the lower number of species of gut bacteria results in, among others, higher intestinal permeability and thus a greater risk of transferring endotoxins into the bloodstream, reduced bile acid signaling, and a lack of short-chain fatty acids (SCFAs). All these adverse effects lead to worsening of fat deposition in the liver, inflammation, and fibrosis (Yang et al., 2025; Qian et al., 2025; Scarpellini et al., 2024; Benedé-Ubieto et al., 2024; Jiang et al., 2025).

Because of this solid causal relationship, research on microbiome-based therapies has gained momentum. Broad microbiome-targeted therapeutic strategies like probiotics, prebiotics, synbiotics, diet adjustments, fecal microbiota transplantation (FMT), and metabolite interventions demonstrate encouraging effects on reducing steatosis, inflammation, and restoration of the intestinal barrier function. On the other hand, the vast majority of evidence is still in the preclinical or early clinical phase. Precision microbiome therapies, combining biomarkers, multi-omics, genetically modified bacteria, and bacteriophages, are another new emerging frontier (Qian et al., 2025). The successful clinical implementation of these precision methodologies critically depends on the establishment of their clinical validity through solid verification, determination of safety parameters, and accurate patient stratification (Zhu et al., 2026; Li et al., 2024; Saeed et al., 2024). Therefore, this review not only aims to put together current evidence regarding the role of the gut-liver axis in MASLD pathogenesis but also evaluates the effectiveness, issues, and future translational potential of microbiome-targeted interventions. Furthermore, while several recent reviews have explored the gut-liver axis (Lau et al., 2025; Zhu et al., 2026; Qian et al., 2025; Schnabl et al., 2025), this review uniquely expands the current literature by contrasting the distinct, stage-specific microbiome signatures between paediatric and adult MASLD populations, an area that remains critically under-researched. Rather than viewing diagnostics and treatments in isolation, this review also integrates novel, multi-omics diagnostic models with precision therapeutic stratification, offering a cohesive roadmap from non-invasive biomarker detection to highly individualized microbiome editing.

## 2 Literature search methodology

To write this narrative review, a comprehensive literature search strategy was performed across all major scientific databases, including PubMed, Scopus, and Web of Science. The search strategy utilized multiple keywords such as “Metabolic dysfunction – associated steatotic liver disease”, “gut-liver axis”, “multi-omics”, and “microbiome-targeted therapeutics”. To capture the rapidly evolving landscape of this field, recently published peer-reviewed papers were considered, although foundational papers from earlier years were also considered, specifically from years 2020–2026.

## 3 Gut dysbiosis and MASLD pathogenesis

One of the major changes resulting from gut dysbiosis in patients with MASLD includes an altered balance between species that leads to intestinal barrier malfunction, endotoxemia, systemic inflammation, insulin resistance, and other pathological changes. A “multiple-hit” or “parallel-hit” concept of pathogenesis, which is supported by current human data and a wealth of literature, has taken the place of the old-fashioned “two-hit” model (Li et al., 2024). This new concept of pathogenesis recognizes gut dysbiosis as a co-factor instead of an independent factor. Actually, various factors such as fat build-up, insulin resistance, microbial metabolites, gene susceptibility, and exposure to the surroundings are involved in a very powerful synergy to trigger the conversion of just fatty liver into fatty liver disease with inflammation and finally fibrosis (Jiang et al., 2025; Zhou et al., 2026). In addition to gut-derived factors, the “multiple-hit” pathogenesis of MASLD is heavily driven by genetic predisposition and the adipose-liver axis. Unhealthy dietary patterns, particularly those rich in glucose, fructose, and saturated fatty acids, promote visceral adiposity and lipodystrophy-like phenotypes (Hrncir et al., 2024). This adipose tissue dysfunction leads to the aberrant release of free fatty acids, ceramides, pro-inflammatory cytokines, and dysregulated adipokines directly into the hepatic circulation. Furthermore, genetic susceptibility plays a pivotal role in mediating this lipid accumulation; specific polymorphisms in genes such as *PNPLA3*, *TM6SF2*, *MBOAT7*, and *GCKR* significantly increase the vulnerability of the liver to steatosis and subsequent metabolic injury. As lipid accumulation and local inflammation progress, the liver actively propagates systemic metabolic dysfunction through the altered secretion of hepatokines, establishing a pathogenic cross-talk with extrahepatic organs. In the pancreas, the elevated hepatic secretion of Fetuin-A, Selenoprotein P, and Follistatin exacerbates beta-cell dysfunction and peripheral insulin resistance, driving systemic hyperglycemia and hyperinsulinemia. Simultaneously, increased circulating levels of liver-derived Angiopoietin-like 3 (Angptl3) strongly contribute to cardiovascular dysfunction and hypertension. Conversely, compensatory endocrine mechanisms are also activated, such as the upregulation of Fibroblast Growth Factor 21 (FGF21), which targets the brain to modulate energy expenditure and central insulin sensitivity. Together, these interconnected pathways illustrate that MASLD operates not merely as an isolated hepatic condition, but as a central driver of systemic, multi-organ disease (Figure 1).

**FIGURE 1:**
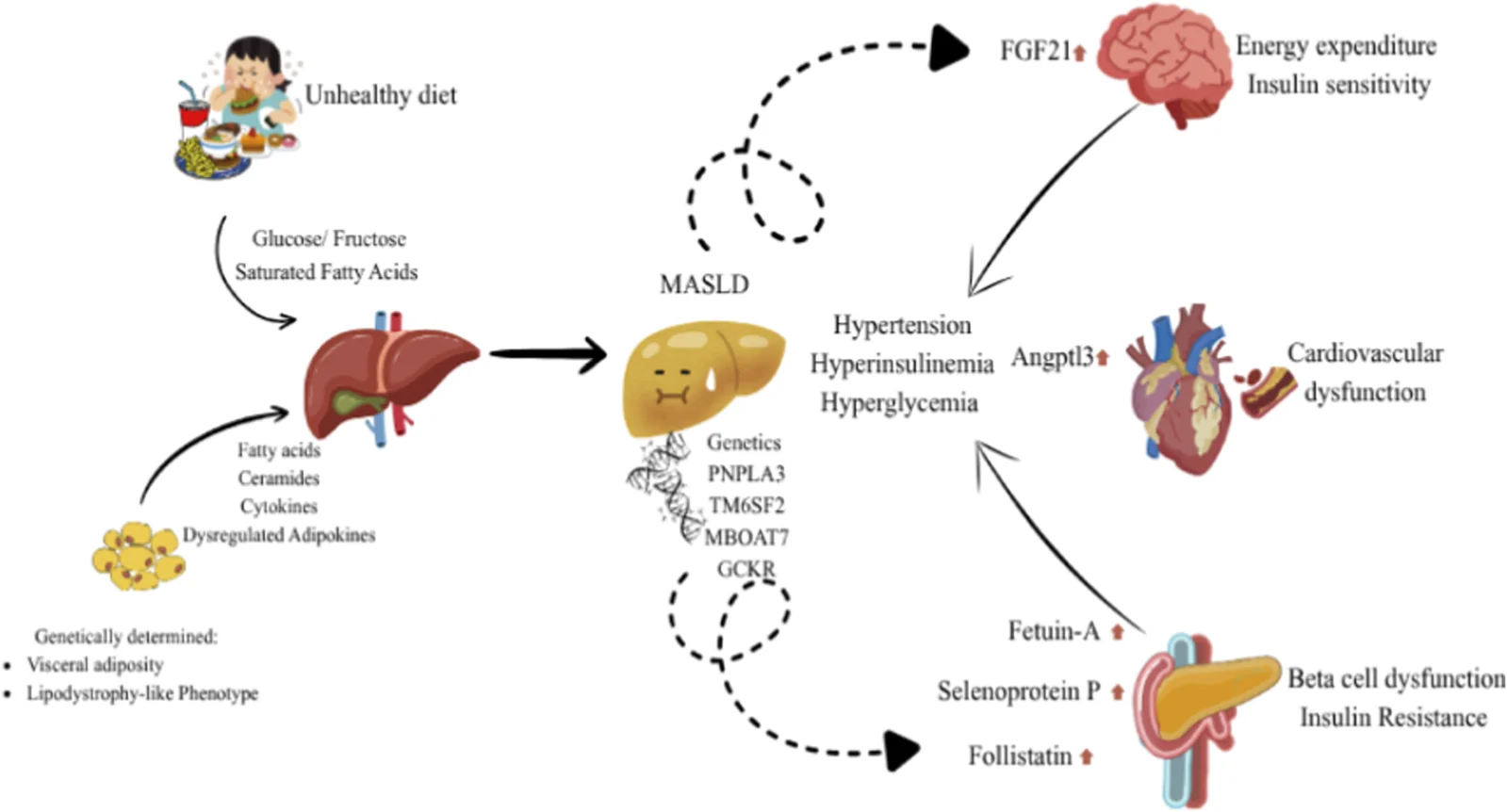
Factors influencing the development of insulin resistance and cardiovascular diseases in association with metabolic dysfunction-associated steatotic liver disease. Abbreviations: Angptl3, Angiopoietin-like 3; FGF21, Fibroblast Growth Factor 21; PNPLA3, Patatin-like phospholipase domain-containing protein 3; TM6SF2, Transmembrane 6 superfamily member 2; MBOAT7, Membrane bound O-acyltransferase domain containing 7; GCKR, Glucokinase regulator.

The main external reasons for this kind of dysbiosis are Western-style diets that normally consist of large amounts of saturated fats, refined carbs, and critically lack fiber. Such dietary habits damage the epithelium and totally overhaul the composition of microbiota that normally reside in the intestines (Termite et al., 2025; Acierno et al., 2025; Ceb et al., 2025). Studies on the subject repeatedly show that there is a significant decrease in microbial alpha diversity and that pathogen-related species are present in abundance in MASLD patients. On the contrary, some of the helpful fiber-degrading species like *Akkermansia* and *Faecalibacterium* have mostly been shown to be depleted (Perazza et al., 2025). As different types of microorganisms are found consistently in MASLD, in many instances it is difficult to pinpoint the precise kind of bacterial change that occurs. (Zhou et al., 2026; Long et al., 2024; Zhou et al., 2025). It has been found in recent integrated studies of all -omics that the bacterial aspect of gut dysbiosis is just a small part of a whole picture involving dysbiosis across different biological kingdoms. These include viral abnormalities, fungal changes, and major metabolic differences such as polyamine and acylcarnitine increases, and reduced secondary bile acid levels (Kim et al., 2025).

Altering the bacterial community in the intestines can lead the liver to develop diseases directly via the gut-liver communication axis. Breakdown of barrier integrity, also known as “leaky gut,” and translocation of bacterial products are mechanisms that connect dysbiosis to liver inflammation (Zhou et al., 2025; Dua et al., 2025). When bacterial products such as lipopolysaccharides (LPS) reach the liver, they provoke oxidative stress and immune system reactions through the TLR4 pathway (Zhang et al., 2024). The oxidative stress caused by bacterial dysbiosis is amplified through ethanol production in our own body, bile acid dysregulation, and mitochondrial failure. All mentioned factors together switch off inflammatory pathways (NF-κB, Nrf2), which are highly sensitive to redox (Scarpellini et al., 2024; Termite et al., 2025). As a result, insulin resistance, which is an important factor in the development of MASLD, is deteriorating and thus contributing to a pathological cycle (Bahitham et al., 2025). In fact, the majority of the published work considers that gut dysbiosis is a crucial but not the only factor for the development of MASLD. This interaction is best described by a multi-way communication and feedback system where dysbiosis, permeability of the gut, endotoxemia, insulin resistance, inflammation, and other related mechanisms play their roles in a constant loop of mutual reinforcement, leading from one to another and finally to the disease.

## 4 Mechanistic links between gut dysbiosis and MASLD

An imbalance between harmful and beneficial gut microorganisms causes gut dysbiosis; it often involves reduced diversity, loss of commensals, or overgrowth of pathobionts. Gut dysbiosis is linked to MASLD mechanistically via 6 recurring pathways, though the strongest consensus is that dysbiosis contributes to MASLD progression through the gut-liver axis (Cui et al., 2025; Schnabl et al., 2025; Abdelhameed et al., 2025; Hamamah et al., 2024) (Figure 2).

**FIGURE 2:**
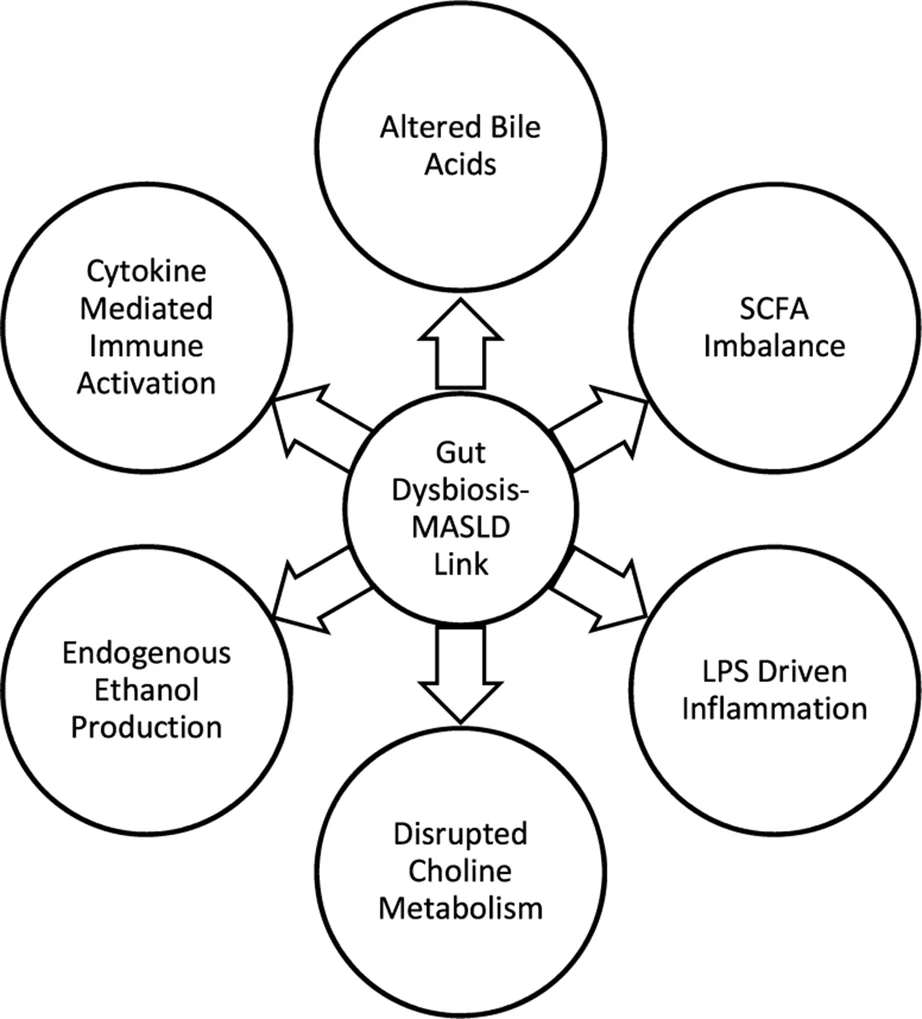
The mechanistic pathways linking gut dysbiosis with MASLD.

Various studies imply that the gut dysbiosis impairs intestinal barrier function and increases permeability and allows microbial products to reach liver and eventually increase MASLD progression and that the dysbiosis changes the microbial metabolites which include bile acids, SCFAs, ethanol, choline related products and TMAO and this further leads to reshaping hepatic lipid metabolism and inflammation along with strong evidences across various studies exhibit that the LPS-TLR4 signalling and cytokine activation are the major inflammatory routes which link gut dysbiosis too steatohepatitis, fibrosis and oxidative stress (Perazza et al., 2025; Long et al., 2024; Luo et al., 2025; Hai et al., 2025). Two major metabolite pathways establish a link between MASLD and Dysbiosis: Altered Bile Acid Metabolism and SCFA Imbalance. The altered bile acid metabolism pathway is one of the clearest links between dysbiosis and MASLD. Gut microbes chemically modify bile acids, and this changes FXR and TGR5 signalling that regulates hepatic glucose metabolism and inflammation (Yang et al., 2025; Li et al., 2024). Recent studies have also established an association between MASLD and bacterial shifts and their involvement in bile acid metabolism, with some fibrosis-linked taxa that suggest stage-specific bile acid dysregulation.

MASLD is linked less to total SCFA absence than to a disturbed balance between SCFA-producing functions and proinflammatory microbial functions. SCFA imbalance appears to matter both because protective SCFA producers are depleted and because SCFA signalling normally supports epithelial integrity and immune restraint. Reviews have consistently established a description for reduced beneficial bacteria, such as Faecalibacterium and Akkermansia, while SCFA-producing bacteria help maintain barrier integrity and reduce inflammation through GPCR signalling (Long et al., 2024; Bahitham et al., 2025; Zazueta et al., 2024).

Furthermore, LPS-mediated inflammation is the best-supported mechanism in the immune system. Dysbiosis weakens the tight junctions and the mucosal barrier, which leads to an increase in translocation of LPS and other microbial components into portal blood, where they activate hepatic TLR pathways (Bahitham et al., 2025; Schnabl et al., 2025). This promotes Kupffer-cell and stellate cell activation, hepatic inflammation, fibrosis, and oxidative stress, with TLR4/NF-kB repeatedly identified as the core signalling route (Bahitham et al., 2025). Immune and cytokine modulation extends beyond LPS alone. Microbial metabolites affect antigen recognition, immune cell recruitment, and pro-versus anti-inflammatory cascades, and dysbiosis is associated with chronic hepatic immune activation rather than a single pathway (Lau et al., 2025; Cui et al., 2025; Abdelhameed et al., 2025). In addition, disruption in choline metabolism contributes by altering microbial processing of choline and related metabolites, which in turn leads to impairment of hepatic lipid export and promotes steatosis (Dua et al., 2025; Luo et al., 2025). Microbiome reviews also report a choline-related metabolite, i.e., TMAO, which directly plays a role in MASLD progression alongside bile acids and SCFAs (Long et al., 2024; Bahitham et al., 2025). Ethanol-producing gut bacteria also provide a toxic route, as endogenous alcohol is also elevated in MASH and is considered a plausible contributor to MASLD pathogenesis (Lau et al., 2025; Li et al., 2024; Luo et al., 2025). Therefore, overall gut dysbiosis contributes to MASLD by disrupting microbial functions, and the most consistent mechanisms are alterations in bile acid and SCFA signalling, barrier breakdown with LPS translocation, and oxidative injury in the liver.

## 5 Microbial signatures and evidence supporting the gut-liver axis in MASLD

Gut bacterial imbalances and the gut-liver axis are among the factors that may contribute to or explain the pathology of MASLD through dysbiosis and microbial signature evidence. The dysbiosis seen is better described through changes at the level of genera, species, and functions rather than single phylum-ratio changes; four major trends have been noted in these changes: fluctuations in the ratio of Firmicutes to Bacteroidetes, disease-associated taxa, developmental differences between children and adults, and the variable effectiveness of microbiome-based diagnostic models. Early reports said the Firmicutes/Bacteroidetes ratio was higher in MASLD and lower in Bacteroidetes. However, more recent pediatric meta-analyses discovered the opposite, i.e., higher Bacteroidetes and lower Firmicutes levels in children with ASH and/or MASLD. Some other populations demonstrated inconsistent phylum-level changes and, more often, elevated Proteobacteria levels. It seems that only the most generalized summaries at the phylum level are not capable of depicting the mechanisms behind the phenomena. More reliable signals are those of the depletion and disappearance of short-chain fatty acid (SCFA) producing microbes, and also an increase in pathogenic or potentially inflammatory microorganisms with fibrosis and steatohepatitis development. A consequence is that describing and diagnosing MASLD has progressively moved away from bacterial proportions to detailed profiles or markers that vary with the age of the patient and the severity of the illness; Notably Table 1 highlights a compelling paradox regarding *Faecalibacterium*, which is consistently depleted in adult MASLD but appears enriched in paediatric cohorts. This age-related discrepancy likely reflects differences in disease duration and the dynamic, still-developing nature of the paediatric microbiome. In children, the enrichment may represent a transient, compensatory microbial response to early metabolic stress or be driven by distinct paediatric dietary habits (such as higher intakes of simple carbohydrates) that select for different microbial profiles. Over time, as the disease progresses into adulthood, the chronic inflammatory environment and prolonged metabolic dysfunction ultimately lead to the depletion and exhaustion of these beneficial, SCFA-producing commensals. In Table 2, we have summarized the outcomes of several attempts to use microbiome profiles in conjunction with other markers (i.e., metabolites, microbial genetic pathways, and host proteins), metagenomics, multi-omics data, and machine learning to come up with noninvasive tests that differ only moderately to highly in accuracy between populations and studies. The identification of these highly specific microbial and metabolic signatures not only aids in non-invasive diagnostics but also provides distinct targets for therapeutic intervention.

**TABLE 1:** Key taxa and age patterns.

Patient population /Disease stage	Enriched taxa/Key markers	Depleted taxa	Ref(s)
Adult MASLD (General)	*Escherichia* Prevotella, Bilophila Veillonella *Streptococcus* Ruminococci-related groups	Faecalibacterium Akkermansia	(Lau et al., 2025; Yang et al., 2025; Li et al., 2024; Lin et al., 2025)
Adult Advanced Fibrosis	*Fusobacterium* *Escherichia* *Shigella*	Lachnospira	(Satthawiwat et al., 2024)
Pediatric MASLD	Prevotella copri Faecalibacterium prausnitzii, Anaerostipes hadrus, Ruminococcus torques	(Not explicitly listed, but distinct from adult patterns with no overlap)	(Luo et al., 2025; Zazueta et al., 2024; Zöggeler et al., 2025)

**TABLE 2:** Diagnostics and functional biomarkers.

Diagnostic Modality/Target	Patient group/Target	Key findings & predictive models	Performance (AUC/AUROC)	Citations
16S Sequencing	Obese Children (MASLD)	Full-length 16S outperformed short-read sequencing for MASLD prediction	86.98% (Full) vs. 70.27% (Short)	(Lin et al., 2025)
Shotgun Metagenomics (ML)	Paediatric Cohorts	Random forest/XGBoost models distinguishing disease stages	87% (MASLD over obesity) 89% (MASH over MASLD)	(Zöggeler et al., 2025)
Taxonomic ML Models	Adult Fibrosis	A three-genus signature distinguished moderate/severe from mild fibrosis	0.93	(Satthawiwat et al., 2024)
Microbiome–Metabolite Model	Adult Fibrosis	Integrated model predicting significant fibrosis	∼0.94	(Ha et al., 2025)
Broad ML Screening	Adult MASLD	General screening for MASLD.	0.93 (0.88 external validation)	(Park et al., 2024)
Metabolites & Pathways	General MASLD	Altered bile acids, SCFAs, TMAO, and tryptophan. Increased alcohol/TMA production; loss of butyrate/methane synthesis	N/A	(Long et al., 2024; Medina-Méndez et al., 2025)
Multi-Omics	Paediatric MASLD	Linked Ruminococcus torques specifically to deoxycholic acid and liver stiffness	N/A	(Du et al., 2025)
Host Protein Markers	Egyptian Adults	TGF-β demonstrated excellent discriminatory accuracy on its own	0.956	(Marawan et al., 2026)
Combined Host/Microbe	MASLD-HCC	Veillonella or host transcript markers combined with AFP at severe progression	0.996 (in one subgroup)	(Jinato et al., 2025)

## 6 Therapeutic strategies targeting gut dysbiosis

### 6.1 Dietary and lifestyle interventions

Dietary behaviours play a foundational role in the prevention and progression of chronic metabolic diseases, wherein high-fat or Western diets induce MASLD together with gut dysbiosis, intestinal barrier disruption, and increased endotoxin/LPS, driving hepatic inflammation and steatosis (Chen K. et al., 2025). Periodontitis worsens MASLD via a microbiota–tryptophan–AHR axis, causing barrier dysfunction, systemic endotoxemia, and mitochondrial stress. Intestinal IL-33 increases barrier permeability, reshapes microbiota (including TMAO-producing bacteria), and aggravates MASLD; IL-33 or ST2 deletion is protective (Chen R. et al., 2025). The gut microbiota is one of the most critical factors in human health. It involves numerous physiological processes impacting host health, mainly via immune system modulation. Changes in microbiota can be multiple, including decreased diversity of species in the gut microbiota, changed metabolic activity, and the occurrence of antibiotic-resistant strains (Forlano et al., 2024; Borges-Canha et al., 2025). Gut dysbiosis is linked to gastrointestinal, metabolic, inflammatory, and neurological diseases; hence, various treatments and therapies aim to restore microbial balance rather than simply suppress symptoms. These strategies range from broad ecosystem resets like diet and FMT to highly targeted microbiome editing. Mediterranean, Japanese, Korean, calorie-restricted, high-polyphenol, high-fibre, plant-based, low-fat, and low-protein diets were all associated with increased abundances of either short-chain fatty acids (SCFA)- or lactic acid-producing bacteria or reduced abundance of opportunistic pathogenic bacteria (Acevedo-Román et al., 2024; Cusumano et al., 2025; Aslam et al., 2026). Hence, fibre-rich diets promote beneficial bacteria and improve dysbiosis-related outcomes (Forlano et al., 2024; Borges-Canha et al., 2025; Wang et al., 2025); calorie-restricted, plant-based, and high-polyphenol diets similarly increase SCFA- or lactic acid-producing microbes and sometimes lower inflammatory markers (Long et al., 2025). Despite significant pharmacological advancements, the rising global prevalence of MASLD necessitates a greater focus on primary prevention. Lifestyle modifications, principally structured to shape gut microbiome health across physical activity, weight management, and sleep/circadian regulation, the evidence is stronger for some links than for others (Sasidharan Pillai and Ashraf, 2026; Zeng et al., 2025; Carmody and Bisanz, 2023; Olson et al., 2026). The clearest patterns are that moderate physical activity is associated with more SCFA-producing taxa, intentional weight loss tends to increase microbial diversity and reduce intestinal permeability, and sleep or circadian disruption is associated with dysbiosis, although causal human evidence remains limited in all three areas. Physical activity tends to modulate the gut microbiota, but the effect depends on exercise type and intensity (Pérez-Prieto et al., 2024; Lin et al., 2024; Zhao et al., 2025). For example, athletes appear to have richer microbiomes than non-athletes, although prolonged high-intensity training can shift the microbiota towards dysbiosis or gut barrier stress (Varghese et al., 2024; Dziewiecka et al., 2022).

Sleep and circadian rhythm regulation appear to be bidirectionally linked with the gut microbiota (Zhang X. et al., 2025; Sahle et al., 2024; Zhang M. Y. et al., 2025). Gut microbes and their metabolites show diurnal rhythmicity, and persistent jet lag, obesogenic diets, or clock disruption can blunt those oscillations, while time-restricted feeding can restore them in experimental work (Sahle et al., 2024). Short sleep and sleep fragmentation are associated with gut dysbiosis and metabolic disturbance. Circadian disruption appears to reduce microbial diversity and expand pro-inflammatory taxa (Table 3) (Gil-Hernandez et al., 2023). Trials of probiotics/prebiotics for sleep remain inconclusive overall, despite some positive individual studies (Mousa and Al Ali, 2024). Overall, lifestyle modifications can influence microbiome health, but the most consistent human evidence supports weight-loss-associated gains in diversity and exercise-associated enrichment of SCFA-related taxa, while sleep and circadian findings are more heterogeneous. The field still needs longer, standardized, multi-omics human trials to determine which microbiome changes are causal rather than correlational (Yuan et al., 2025).

**TABLE 3:** Summary of current research exploring the effects of behavioral and short-term weight loss interventions on the gut microbiome, highlighting main findings, key microbial shifts (e.g., Akkermansia), and current evidence limitations.

Topic	Main finding	Limitation	Ref(s)
Behavioral weight loss	Alpha-diversity increases and intestinal permeability falls with intentional weight loss	Evidence is imprecise despite consistent direction	(Koutoukidis et al., 2022)
Specific taxa	Akkermansia increases most consistently with weight loss	No clear evidence for broad phylum-level shifts or fecal SCFA changes	(Koutoukidis et al., 2022)
Short-term intervention	3-week energy restriction plus vigorous exercise improves body composition and insulin sensitivity without changing the gut microbiome	Early metabolic benefits were unlikely microbiome-mediated	(Aoun et al., 2020)
RCT synthesis	Most obesity-targeted interventions did not significantly change alpha or beta diversity	Prebiotics and probiotics showed more consistent taxonomic shifts than lifestyle-only interventions	(Lee et al., 2026)

### 6.2 Microbiome-targeted therapeutics

In MASLD patients, specific microbial signatures correlate with steatosis and fibrosis severity, including enriched potentially pathogenic genera and reduced beneficial taxa (Satthawiwat et al., 2024; Forlano et al., 2024; Borges-Canha et al., 2025). Probiotics like *Lactobacillus* rhamnosus GG and other mixtures improve steatosis, inflammation, barrier integrity, and SCFA production in MASLD, MASH, or MASLD-HCC models (Benedé-Ubieto et al., 2024; Wang et al., 2025; Zhang et al., 2026). Early animal work in “dual-hit” SLD shows partial improvement in liver and gut phenotype after FMT. Microbial features like Bact2 enterotype, specific genera, etc., markedly improve diagnostic models for severe steatosis beyond clinical scores alone (Borges-Canha et al., 2025). Clinical evidence for FMT in MASLD presents a mixed but evolving picture. For instance, a randomized controlled trial by Xue et al. (2022) (Xue et al., 2022) compared standard oral probiotics against heterologous FMT (administered via colonoscopy followed by three subsequent enemas). At the 1-month follow-up, the FMT cohort demonstrated a significant decrease in hepatic fat accumulation and successfully reconstructed gut microbiota, with the therapeutic efficacy notably higher in lean MASLD patients compared to obese MASLD patients. Conversely, a recent double-blind RCT by Groenewegen et al. (2025) (Groenewegen et al., 2025) evaluating the effect of three consecutive FMTs in 20 MASLD patients found a null outcome. At the 12-week primary endpoint, there were no significant changes in hepatic steatosis (as measured by MRI-PDFF), glucose tolerance, or liver biochemistry between the treatment and placebo groups. These contrasting results underscore that while FMT has the potential to modulate the gut-liver axis, its clinical translation is heavily dependent on administration protocols, patient phenotypes, and the specific metrics used to evaluate liver fat. Animal and human data jointly support that the gut pathology directly triggers or worsens the liver pathology in MASLD. The diet and host factors alter the microbiota, compromise the intestinal barrier, and increase hepatic inflammation and fibrosis.

Probiotics, prebiotics, and FMT are examples of microbiome-targeted therapeutics that modulate the gut microbiota and achieve beneficial effects for the host (Airola et al., 2023; Chen K. et al., 2025).

Fecal microbiota transplantation (FMT) has been investigated as a procedure to restore the equilibrium of the gut microbiota, as explained in Tables 4, 5. It has shown benefits in many diseases, including inflammatory bowel disease, chronic liver disorders, and extraintestinal autoimmune conditions (Airola et al., 2023). FMT restores community structure and is established for recurrent *C. difficile*, with expanding use in IBD, metabolic, and liver disease (Sahle et al., 2024; Airola et al., 2023; Farah et al., 2025). Antibiotics can both treat dysbiosis-related infections and cause dysbiosis; narrow-spectrum or “eubiotic” antimicrobials like rifaximin are being explored as microbiota-sparing antibiotics (Acevedo-Román et al., 2024; Cusumano et al., 2025). While promising, their clinical translation requires rigorous safety assessments to address concerns regarding horizontal gene transfer and long-term ecological impact (Zhang M. Y. et al., 2025).

**TABLE 4:** Comparison of clinical effects and use cases across different types of microbiome-directed therapies.

Biotic type	Primary clinical outcomes/Effects	Target conditions/Use cases	Citations
Probiotics	Increases clinical remission rates Reduced pro-inflammatory markers Helps restore microbial balance	Inflammatory Bowel Disease (IBD) Antibiotic-induced dysbiosis (microbial imbalance) Gut-brain axis disruptions	(Cusumano et al., 2025; Farah et al., 2025)
Prebiotics	Increases clinical remission rates Reduced pro-inflammatory markers	Inflammatory Bowel Disease (IBD)	(Farah et al., 2025)
Synbiotics (Probiotics + Prebiotics)	Increases clinical remission rates Reduced pro-inflammatory markers Helps restore microbial balance	Inflammatory Bowel Disease (IBD) Antibiotic-induced dysbiosis (microbial imbalance) Gut-brain axis disruptions	(Cusumano et al., 2025; Dahiya and Nigam, 2023)
Postbiotics (Microbial Metabolites)	Provides a safer, highly stable treatment option	Metabolic Disorders	(Ahmed et al., 2025)

**TABLE 5:** Microbiome therapies: aims, evidence, and applications.

Strategy	Aim	Evidence/context	Citation
Mediterranean/high fibre diet	Enrich SCFA producers, reduce inflammatory markers	Sometimes lower inflammation	(Aslam et al., 2026)
Probiotics/synbiotics	Restore diversity, support barrier, reduce inflammation	Higher remission in IBD and helps after antibiotics	(Cusumano et al., 2025; Farah et al., 2025)
FMT	Whole ecosystem reset	Proven in recurrent CDI, benefits in IBD and metabolic disease, but safety/standardisation issues	(Sahle et al., 2024; Farah et al., 2025)
Engineered Bacteria	Deliver therapeutic molecules or missing functions	Next-generation probiotics for IBD and other diseases	(Zhang M. Y. et al., 2025)

### 6.3 Next-generation and precision interventions

The use of engineered probiotic bacteria or bacteriophage-based therapy, have recently appeared as promising strategies to provide targeted and tailored therapeutic modulation of the gut microbiota Phage therapy can also selectively deplete pathobionts (Pathobionts are typically harmless or beneficial microbes that are native to the body’s microbiome that turn harmful under specific genetic or environmental conditions.), early trials show targeted reduction of *fusobacterium* nucleatum and improved cancer therapy response. Upcoming and emerging tools for gut dysbiosis include engineered probiotics, synthetic consortia, phage cocktails, targeted metabolite or RNA delivery, and precision microbiome medicine guided by multi-omics and AI. Combination regimens such as probiotics + Mediterranean diet + FMT may have synergistic effects on mucosal healing (Acevedo-Román et al., 2024; Airola et al., 2023; Sahle et al., 2024; Mousa and Al Ali, 2024).

To address the therapeutic gaps of current standards of care, a robust pipeline of active clinical trials is presently evaluating novel interventions. The new microbiome-targeted treatment for MASLD is a promising but early-stage field: probiotics, prebiotics, synbiotics, FMT, dietary microbiome modulation, and next-generation live biotherapeutics are all under study, but few interventions have robust MASLD-specific clinical evidence yet (Benedé-Ubieto et al., 2024; Schnabl et al., 2025). The field is also shifting from broad microbiome restoration toward precision ecosystem editing based on metabolites, barrier dysfunction, and patient-specific microbial profiles (Qian et al., 2025; Zhou et al., 2026). Few studies claim that probiotics, prebiotics, and synbiotics are the most actively studied microbiome-based MASLD interventions, with promising signals for improving hepatic steatosis, inflammation, gut permeability, and microbial balance, but the evidence base is still dominated by small or heterogeneous studies rather than standardized large trials; FMT appears to improve liver or metabolic parameters in some MASLD/MASH studies, and current microbiome-based therapy development spans dietary modulation, probiotics, prebiotics, synbiotics, FMT, and metabolite-focused approaches rather than a single dominant platform. Precision strategies increasingly aim to match therapy to the dominant dysfunction in each patient, such as bile acid dysregulation versus barrier dysfunction, using microbiome or metabolite profiling and non-invasive endpoints. Reviews also highlight phage-guided depletion, engineered bacteria that deliver therapeutic molecules, fungal or microbial protective metabolites, and AI-assisted multiomics stratification as likely next-wave translational platforms (Zhu et al., 2026; Qian et al., 2025; Zhou et al., 2026; Perazza et al., 2025; Schnabl et al., 2025).

The main barriers to translation are standardization, scalability, and personalization. Overall, targeting gut dysbiosis in MASLD is advancing from broad modulation toward precision microbiome therapeutics, but current research and clinical trials still support cautious optimism rather than established clinical use.

## 7 Challenges and future directions in MASLD

Integrating microbiome-centered therapies into routine clinical practice for MASLD still faces considerable barriers, driven largely by patient heterogeneity and unresolved regulatory safety concerns (Marawan et al., 2026; Zhou et al., 2026). Because individual differences in sex, lifestyle, ethnicity, age, and genetics create diverse MASLD sub-phenotypes, therapeutic microbial patterns found in one demographic rarely apply directly to another (Pirola et al., 2024). This variability is further complicated by the field’s heavy reliance on single-time-point cross-sectional studies, which makes it difficult to track long-term microbial shifts or accurately predict how an individual will respond to a specific therapy (Bahitham et al., 2025). For decades, MASLD management was largely restricted to lifestyle modifications like diet and exercise (Younossi et al., 2024; Yang et al., 2025; Scarpellini et al., 2024; Zhou et al., 2026; Rozera et al., 2025). However, the recent advent of approved pharmacotherapies has fundamentally altered this clinical landscape. In March 2024, the FDA granted accelerated approval to resmetirom (Rezdiffra) for non-cirrhotic MASH with F2-F3 fibrosis (Harrison et al., 2024), followed by the accelerated approval of semaglutide 2.4 mg (Wegovy) in August 2025 for the same patient population based on the ESSENCE phase 3 trial (Sanyal et al., 2025). With these medications now available, microbiome-targeted interventions need to be contextualized differently. Going forward, they are better positioned as adjunctive or combinatorial strategies that can synergize with approved drugs to further support mucosal healing and metabolic recovery. At the same time, broad regulatory approval for interventions like FMT and phage therapy is still limited. Bringing these therapies into the clinic is held back by a lack of standardized protocols for donor selection, fecal sample preparation, and dosing, along with genuine safety concerns regarding gastrointestinal adverse events and accidental pathogen transmission (Zhang M. Y. et al., 2025). Overcoming these obstacles will require the field to establish consensus methodologies and conduct rigorous, long-term clinical validation (Schnabl et al., 2025). Ultimately, the ability to accurately predict treatment success relies on advancing precision medicine. By leveraging multi-omics and AI-guided patient stratification, researchers can integrate microbiome, genetic, and clinical data to move beyond broad ecosystem resets and design therapies uniquely tailored to the individual.

## 8 Conclusion

The escalating global prevalence of MASLD makes it abundantly clear that traditional, lifestyle-only management is no longer sufficient to curb this chronic health challenge. As explored in this review, the gut-liver axis is not merely a passive bystander in liver disease; rather, it is an active, dynamic engine driving MASLD progression. When the delicate equilibrium of the gut microbiome is disrupted, the resulting cascade of intestinal barrier dysfunction, systemic endotoxemia, and metabolic shifts, such as altered bile acid and short-chain fatty acid signaling, directly fuels hepatic steatosis, inflammation, and fibrosis. Fortunately, recognizing this profound bidirectional relationship has unlocked a promising new therapeutic frontier. Broad microbiome-targeted interventions, including dietary modifications, biotics, and fecal microbiota transplantation (FMT), have demonstrated genuine potential to restore microbial balance, heal the mucosal barrier, and ease the metabolic burden on the liver. However, translating these early successes into routine clinical practice is currently hindered by the immense heterogeneity of patient microbiomes and a lack of standardized clinical protocols. The evidence highlights that when it comes to the microbiome, treatment cannot be a one-size-fits-all endeavor. Looking ahead, the next major leap in MASLD care will rely on a transition from broad ecosystem resets to targeted precision medicine. By harnessing the power of multi-omics, AI-guided patient stratification, and next-generation tools like engineered biotherapeutics and bacteriophage therapy, clinicians will eventually be able to map a patient’s specific microbial deficits and deliver highly tailored interventions. While rigorous, longitudinal clinical trials are still urgently needed to ensure safety and long-term efficacy, the path forward is clear: the future of treating the liver will increasingly depend on precisely healing the gut.
